# Association Between the Predominant Domain of Eating Behavior and Perception of Distortion and Satisfaction with Body Image in People Who Underwent Bariatric Surgery in Brazil

**DOI:** 10.3390/nu17050850

**Published:** 2025-02-28

**Authors:** Renata Cristina Bezerra Rodrigues, Izabella Syane Oliveira Pereira, Gizeuda Rosi Bahia, Alvaro Lucas Fernandes Souza, Paula Raimunda Araújo Teixeira, Carla Cristina Paiva Paracampo, Naíza Nayla Bandeira de Sá

**Affiliations:** 1 Carlos Chagas Filho Biophysics Institute, Federal University of Rio de Janeiro, Rio de Janeiro 21941-971, Brazil; 2 Center for Theory and Research on Behavior, Federal University of Pará, Belém 66075-110, Brazil; izabella.o.pereira@gmail.com (I.S.O.P.); cparacampo@gmail.com (C.C.P.P.); naizasa@ufpa.br (N.N.B.d.S.); 3 Institute of Health Sciences, Federal University of Pará, Belém, 66075-110, Brazil; gizeudarosi@hotmail.com; 4 Faculty of Nutrition (FANUT), Federal University of Pará, Belém 66075-110, Brazil; alvarolucasfs@gmail.com (A.L.F.S.); paula.araujot@gmail.com (P.R.A.T.)

**Keywords:** bariatric surgery, body dissatisfaction, body image, eating behavior

## Abstract

Background and Aims: Bariatric surgery is an effective treatment for weight loss and improvement of associated comorbidities. However, some factors could negatively influence favorable results after surgery. This paper aimed to identify whether there is an association between the predominant domain of eating behavior and perception of distortion of and satisfaction with body image in people who underwent bariatric surgery in Brazil. Materials and Methods: To this end, we carried out a cross-sectional, quantitative, analytical study with a convenience sample and with data collected through online questionnaires available on Google Forms™. Results: There was a higher frequency for females. Cognitive restriction was the predominant eating behavior domain. We found an association between body image distortion and cognitive restriction (*p* = 0.001) and between body image distortion and emotional eating (*p* = 0.001). Conclusions: The results elucidated the importance of researching eating behavior and body image in people who have had bariatric surgery. New prospective studies should be encouraged to understand the cause–effect relationship between eating behavior and body image.

## 1. Introduction

Obesity is a chronic, non-communicable condition with high morbidity and mortality rates and constant growth in incidence rates [1]. To reduce morbidity, weight loss is essential, and it can be achieved with lifestyle changes, medicines, and behavioral therapies [2]. However, for severe obesity or failure of initial treatments, bariatric surgery (BS) is considered the most effective treatment [3], with improvement in comorbidities [4] and reduction of mortality due to associated comorbidities [5].

Currently, in contrast to other countries, in Brazil, Roux-en-Y gastric bypass is the most common technique [6,7,8], and it is considered the gold-standard technique, with substantial results in the sustained reduction of body weight [2,4,8,9]. Moreover, the benefits of BS can be seen in improving hypertension, dyslipidemia, and type 2 diabetes. Nevertheless, weight loss is usually the preferred variable when analyzing results, especially for patients [10].

Gradually recovering up to 10% of the excess weight lost, without clinical harm, is considered normal [11]. However, weight regain greater than 10% is positively associated with dysfunctional eating behavior, a precursor to eating disorders, such as binge eating disorder [3,12,13]. Worsening matters, dissatisfaction with and distortion of body image after BS are recurrent and related to more frequent episodes of dysfunctional eating behavior [14,15].

Dissatisfaction with body image appears more frequently in the early stages of the postoperative period, improving between 6 and 24 months, with subsequent reduction. The perception of the image of a person who goes from an “obese” body to a “thin” one occurs more slowly than weight loss [16,17]. Several factors influence these results, but they have not yet been fully understood [15].

Although the findings are relevant and suggest a possible correlation between eating behavior and body image, such detail has not yet been verified in people who have undergone bariatric surgery.

Thus, this research aimed to identify (a) the predominant dysfunctional eating behavior; (b) the degree of satisfaction with and distortion of body image; and (c) whether there is an association between the three domains of eating behavior (uncontrolled eating, emotional eating, and cognitive restraint) and the degree of distortion of and satisfaction with body image in people who underwent bariatric surgery in Brazil.

## 2. Materials and Methods

This was a cross-sectional, quantitative, analytical study, with a non-probabilistic sample obtained through convenience sampling. This was a convenience sample focusing on a specific population, which was people who underwent bariatric surgery. Convenience sampling can facilitate the inclusion of populations that would be difficult to reach otherwise, allowing for the collection of data on underrepresented groups.

Convenience sampling can introduce biases that limit the generalization of the results, but generalizing the results to the rest of the population is not part of our objectives.

The research was carried out using self-administered online questionnaires available on Google Forms™.

In order to achieve a significant sample of the group selected for the study, patients who underwent BS, we performed a sample calculation based on the number of bariatric surgeries performed in Brazil according to data recorded by DATASUS (the Department of Information Technology of the Unified Health System). In 2019, 12,569 procedures performed by the Unified Health System (SUS) were recorded [7].

Therefore, for our study design, we considered as statistical parameters the total population size of 12,569 patients, a 95% confidence level, and a 5% margin of error. To determine n, we used a free online sample calculator (available at https://pt.surveymonkey.com/mp/sample-size-calculator/ accessed on 6 May 2020), resulting in an approximate number of 385 participants.

In total, 398 people who underwent bariatric surgery participated in this study by signing the Free and Informed Consent Form (ICF). At the end of the ICF, only the “I agree” option allowed access to the questionnaires. The “I disagree” option ended participation, taking the participant to the final screen with a thank you message.

All questions were marked as mandatory, and only a completely answered questionnaire gave access to the next one. Exclusion criteria were people who had repeated bariatric surgery, people who did not have access to the internet to fill out the questionnaires online, and people who lived outside of Brazil.

The questionnaires were presented to the participants in the following order: (1) ICF, (2) questionnaire on sociodemographic and anthropometric profile and information on the surgical procedure, (3) the Three Factor Eating Questionnaire (TFEQ-21), (4) the Body Shape Questionnaire (BSQ), and (5) the Silhouette Scale by Stunkard et al. [18] (1983).

For BMI values, we used the weight (kg) and height (cm) data requested in the sociodemographic questionnaire and the standard BMI formula of BMI = height (m)/weight^2^ (kg). We requested the weight before surgery for the “preoperative nutritional status” data, the current weight for “current nutritional status”, and the lowest weight achieved for “lowest BMI achieved”, using the same height value to calculate the three BMI values.

The TFEQ-R21 consists of 21 items, with four response options on a Likert scale. The objective is to track three dysfunctional eating behaviors: uncontrolled eating, emotional eating, and cognitive restraint. Among the three, the one with the highest score is the predominant domain. The final score was obtained according to the classification instructions provided by the authors [19].

The BSQ consists of 34 sentences, with six responses on a Likert scale. The objective is to track the presence of some degree of body image distortion between no distortion, mild distortion, moderate distortion, and severe distortion. The final score was obtained according to the classification instructions provided by the authors [20].

The Silhouette Scale developed by Stunkard et al. [18] (1983) is formed by a set of nine figures of females and males, representing human silhouettes according to the increasing average of the Body Mass Index (BMI). The numerical difference between the figure chosen as the current real image and the figure chosen as the desired image, selected on the scale, represents the degree of satisfaction with body image. The final score was obtained according to the classification instructions provided by the authors [18].

To assess the normality of the variables, the Shapiro–Wilk test was used. For the descriptive analysis, the absolute and relative frequencies of all study variables were calculated according to the duration of surgery (0 to 24 months and 25 months or more) and the total. To test associations between the domains of eating behavior and the degree of body image distortion and satisfaction with body image, the univariate prevalence ratio was calculated using log-linear Poisson regression, considering 95% CI and *p* ≤ 0.05.

The reference categories adopted to calculate the prevalence ratio were those with the greatest protection for each of the three domains of eating behavior. Association was considered when *p* ≤ 0.05. Data analyses were performed using the Statistical Package for the Social Science (SPSS) version 17.0 for Windows.

## 3. Results

### 3.1. Sociodemographic Data

Among the sociodemographic characteristics, there was a greater frequency of females (93.5%) between 40 and 59 years old (57.3%) who had bariatric surgery 25 months prior or more (52%) with a marital status of married or in a stable union (60.8%) and an education equal to or greater than 12 years of study (81.2%), which represents incomplete/complete higher education (Table 1).

### 3.2. Pre- and Postoperative Data from Bariatric Surgery

Roux-en-Y gastric bypass appeared as the most common surgical technique (80.2%). Regarding nutritional status, a higher frequency of grade III obesity (57%), followed by grade II (38.9%), was observed preoperatively. In the current nutritional status, the highest frequencies were overweight (36.9%), grade I obesity (25.6%) and normal weight (23.1%). The lowest BMI achieved for most of the sample was eutrophic (36.5%) and overweight (36%) (Table 2).

### 3.3. Satisfaction with and Distortion of Body Image and Eating Behavior

The results of the Silhouette Scale showed 87.5% dissatisfaction with body image, with the most frequent desire for a smaller silhouette (82.2%). In the Body Shape Questionnaire, 38.4% showed some degree of body image distortion. The predominant domain of the eating behavior with higher frequency was cognitive restriction (CR) in 75.9% of the sample, followed by emotional eating (EE) with 15.3% (Table 3).

### 3.4. Association Between the Predominant Domain of Eating Behavior and Body Image

#### 3.4.1. Emotional Eating as the Predominant Domain (EE-PD)

In column 1, some degree of body image distortion is observed in 65.5% of respondents with emotional eating as the predominant domain (PD). When compared with people from the same group with no distortion, people with moderate (RP 6.32), severe (RP 4.44), and mild (RP 2.74) body image distortion were more likely to present EA as a predominant domain (EE-PD), with a statistically significant association (*p*-value = 0.001).

In the Silhouette Scale, it was observed that 91.8% of people with EE as the predominant domain expressed dissatisfaction with their body image, and, of these, 88.5% had a desire for a smaller silhouette. There was no statistical association between EE and satisfaction with body image (*p*-value = 0.336) (Table 4).

#### 3.4.2. Uncontrolled Eating as the Predominant Domain (UE-PD)

In column 2, it is observed that 47.4% of people with uncontrolled eating as the predominant domain (UE-PD) showed no distortion, 42.1% showed mild distortion, and 100% showed dissatisfaction with body image. In this same group, there was the highest frequency of desire for a smaller silhouette (UE-PD 94.7%) when compared to the other domains of emotional eating (EE-PD 88.5%) and cognitive restriction (CR-PD 79.7%). No statistical associations were found between UE and distortion (*p*-value = 0.064), nor with satisfaction with body image (*p*-value = 0.23) (Table 4).

#### 3.4.3. Cognitive Restriction as the Predominant Domain (CR-PD)

In column 3, it is observed that 69.8% of participants with cognitive restriction as the predominant domain (CR-PD) showed no body image distortion. There was a significant statistical association (*p* = 0.001) between CR-PD and body image distortion, where people who presented mild (RP 0.29), moderate (RP 0.19), or severe (RP 0.19) image distortion were less likely to present with CR as the predominant domain when compared to people from the same group with no distortion.

Still in CR-PD, 85.4% of participants showed dissatisfaction and 14.6% showed satisfaction with body image, which is a higher frequency of satisfaction compared to the other domains (EE-PD = 8.2% and no percentage for UE-PD). However, no statistical association was found between cognitive restriction, such as PD, and satisfaction with body image (*p* = 0.064).

## 4. Discussion

The main findings of this study revealed that cognitive restriction was the most frequent domain of dysfunctional eating behavior among participants (75.9%). There was a statistical association between eating behavior and body image distortion, but not between eating behavior and dissatisfaction with body image.

Regarding the sociodemographic profile of the sample, there was a higher frequency of females. A significant majority of female participants is a recurring result in studies on bariatric surgery (4.21–24). The justifications presented in studies vary, such as greater personal motivation for weight loss, greater female concern with aesthetics and/or health, and greater frequencies of dissatisfaction with body image.

In the sample, the majority of volunteers presented cognitive restraint as the predominant domain, followed by emotional eating and uncontrolled eating.

Eating disorders can be seen in other similar studies. Tan et al. [5] (2021) verified the eating behavior of BS patients at three moments: preoperatively and six and 12 months after surgery. Preoperatively, there was a predominance of uncontrolled eating and emotional eating, with a reduction in scores at 6 months and a slight increase at 12 months. Meanwhile, cognitive restriction showed significant increases at the three moments [21].

The clinical trial by Engström et al. [22] (2015) separated people who had bariatric surgery into two groups according to the result of uncontrolled eating on the TFEQ-R21: good eating control (GEC) and poor eating control (PEC). Differences between groups were large even before surgery, but they increased significantly at the 2-year follow-up. In the PEC group, significantly more dysfunctional eating behaviors were observed, with improvements after 1 year and a return to baseline levels after 24 months. In the GEC group, there were significantly favorable differences, which remained stable even after 2 years.

Postoperative uncontrolled eating has been associated with a greater risk of losing control over eating when feeling hungry [23] or when exposed to external stimuli [22] and frequent eating, also called grazing behavior, especially of high-calorie foods [24]. Consequently, this negatively affects weight loss and maintenance of weight lost after BS [22,23,25,26,27]. In studies with longer follow-up, emotional eating predicted worse weight loss and was related to weight regain at 48 months [26].

Within this context, it is noted that bariatric surgery produces broad physiological and behavioral effects that impose changes in diet such that eating behavior becomes even more complex [2,23,25]. Thus, tracking predominant dysfunctional eating behavior before surgery is an attempt to prevent related consequences [26].

In addition to weight loss, faced with the change in image resulting from weight loss, image distortion and dissatisfaction with body image are recurrent and have modifiable degrees. Among our participants, we found dissatisfaction with body image in 87.2% and distortion with body image in 38.4%.

In the study by Silva et al. [17] (2020), the highest frequency in the sample was for the presence of some degree of body image distortion at 55.56%, with 25% being moderate and 5.6% being severe distortion. In the same study, in the Silhouette Scale, there was a high frequency of body image dissatisfaction, with 83.3% of patients wanting to reduce their silhouette.

Other studies that used the Silhouette Scale also presented similar results. Caldeira et al. [28] (2020) found that 95.4% of participants felt dissatisfaction with their body image and 90.7% had a desire for a smaller silhouette. Lacerda et al. [16] (2018) found that 85.3% of women and 50% of men had image dissatisfaction and a desire for a smaller silhouette. These results corroborate the findings in the present study, highlighting the importance of studies on body image.

In the tests carried out between the main research variables, there was a statistical association between emotional eating (EE) and cognitive restriction (CR) as the predominant domain and body image distortion. This result revealed that people with image distortion were more likely to develop EE and less likely to develop CR, according to prevalence ratios.

Higher CR frequencies have been associated with favorable results [22,26] due to the direct relationship of presence of dietary restrictions → weight loss → procedure success. The presence of CR in the postoperative period is expected due to the physical and hormonal changes that surgery causes in individuals [2,29]. However, there is a fine line that needs to be carefully monitored so that this dysfunctional behavior is not strengthened to the point of triggering restrictive eating disorders, such as anorexia nervosa [10,13].

A qualitative study conducted by Watson et al. [10] (2020) sought to understand the reasons related to excessively restrictive eating behaviors in women after BS. The analysis revealed three common themes: (1) weight stigma, where high levels of weight bias increase the risk for restrictive eating disorders, (2) excess skin as a reminder of lost weight, a condition that can evoke fear of weight gain and excessively restrictive eating behaviors, and (3) disordered thoughts and eating patterns, which can generate anguish, guilt, and intensified fears regarding weight gain.

It is worth noting that the spatial limitation of gastric reduction already imposes a restrictive diet and limited postoperative energy intake on the patient. In addition, BS results in significant reductions in resting metabolic rate. However, food intake must meet the patient’s daily protein–calorie needs to keep him/her nutritionally healthy throughout the postoperative period, thus requiring avoidance of excessively restrictive eating behaviors [30].

Studies on body image distortion, aggravated by excess skin, show a link with physical discomfort, significant impairment in quality of life, psychosocial problems, and social isolation, which are also motivated by feelings of image shame [10,23,27]. de Lourdes et al. [27] (2021) studied factors related to excess skin in women and observed that 81% of them reported discomfort in one or two areas of the body, and this discomfort was correlated with body image shame, suffering psychologically, and lack of eating control.

Finally, satisfaction with body image, assessed using the Silhouette Scale, showed frequent dissatisfaction with body image and the desire for a smaller silhouette. However, no associations were found between image satisfaction and the domains of eating behavior. A possible explanation for the non-association may be the non-specificity of the Silhouette Scale to assess body image in the postoperative period of bariatric surgery.

The use of Silhouette Scales must be judicious, and special attention must be given to the use of appropriate scales for each population [31]. There are no instruments capable of assessing dissatisfaction with body image in the postoperative period of bariatric surgery that assess specific issues, such as excess skin [32,33].

Dias [34] (2014) also states that the lack of representation and drawings far from reality are considered weaknesses of Silhouette Scales. Therefore, it is suggested that new studies on body image, including, specifically, those that assess satisfaction/dissatisfaction with the size of body image, be developed with specific tools for people postoperatively after bariatric surgery.

Our findings highlight the importance of multidisciplinary follow-up for the maintenance of long-term bariatric surgery outcomes. This monitoring involves the collaboration of several health professionals, such as doctors, nutritionists, psychologists, and physical educators. However, not all patients have the resources available to pay for these health professionals.

Therefore, it is worth noting that public policies can guide possible health and obesity-fighting interventions, encompassing this segment of the population that, as the data presented in this research indicate, shows annual growth, including in the public health system. Public policies play a fundamental role in promoting effective treatment and ensuring access to multidisciplinary monitoring.

Although the benefits of a multidisciplinary team are proven in the literature, there are still challenges in implementing this monitoring. The lack of professionals specialized in this topic, especially in some regions, the scarcity of public health resources, and the resistance of patients to seeking continuous support can limit the effectiveness of this treatment.

Initiatives, such as the creation of referral centers for the treatment of obesity and the formation of multidisciplinary teams in health units, can improve the scenario. Integration between the different areas of health is essential to promote the recovery and quality of life of patients.

The methodology used in data collection enabled the participation of people from all regions of Brazil. The use of self-administered online questionnaires allowed for total flexibility in the time and place for completion, thus facilitating participation in the study.

Some limitations are inherent to this type of self-reported data, such as the omission of information, under- or overestimated data, and other limitations of research that requires antecedent data and where the participant’s memory bias can influence the accuracy of the results [12].

The use of a convenience sample may not be representative of the entire bariatric patient population. However, given the nature of our study, our research objectives, and the characteristics of our population, we found convenience sampling to be an effective way to generate our data.

The use of self-administered questionnaires may have introduced response bias or underestimation of data. Some limitations are added to this type of self-reported data, such as omission of information, under- or overestimation of data, and other research limitations that require previous data, where participant recall bias may influence the accuracy of the results. However, self-administered questionnaires can be safe and effective in epidemiological research, as long as they are validated and have participant compliance.

A strength of our study is the use of validated psychometric tools that accurately measure what they are intended to measure (validity) and that produce consistent results over time (reliability), such as the TFEQ-21 and BSQ questionnaires. Choosing validated tools facilitates statistical analysis as they have standardized methods, which not only strengthens the quality of the data collected but also enables real comparison between results from other studies.

The cross-sectional nature of the study does not allow for determining causality. Cross-sectional studies allow for analysis of possible correlations between variables in a given population at a specific time, are they are low-cost and quick to carry out. However, they do not allow for understanding cause and effect between variables. In the case of this study, it is not possible to conclude whether the lack of control over eating occurred first and then the distortion of body image or the opposite. Therefore, we suggest carrying out new longitudinal studies with people who have had bariatric surgery to evaluate eating behavior and body image over a period of time.

## 5. Conclusions

The predominant domain of dysfunctional eating behavior was cognitive restriction. The findings revealed that there was a greater frequency of some degree of image distortion, as well as a greater desire for a smaller silhouette. In the associations tested, we found that people with some degree of image distortion were less likely to have cognitive restriction as the predominant domain and more likely to have emotional eating as the predominant domain.

Tracking and identifying dysfunctional eating behaviors and knowing the predominant domain in different situations offer more possibilities for intervention and individual monitoring. Furthermore, other psychological factors not considered in this study may be useful for producing broader and more efficient knowledge, such as a more in-depth exploration of variables, such as self-esteem or anxiety.

These results provide useful information for multidisciplinary monitoring. However, further longitudinal and prospective studies are recommended and may increase the validity of the results found.

## Figures and Tables

**Table 1:** Sociodemographic factors of people who underwent bariatric surgery in Brazil.

Variables	n	%
Gender		
Female	372	93.5
Male	26	6.5
Age group *		
From 20 to 39 years	170	42.7
From 40 to 59 years	228	57.3
Time post-surgery **		
From 0 to 24 months	182	45.7
≥25 months	207	52
Marital status		
Married/stable union	242	60.8
Divorced/single/widower or widow	156	39.2
Education (years)		
From 0 to 8	6	1.5
From 9 to 11	69	17.3
More than 12	323	81.2

* The youngest age found was 20 years old. ** Considering 9 volunteers who did not correctly answer the question about bariatric surgery time.

**Table 2:** Pre- and postoperative characteristics of bariatric surgery.

Variables	n	%
Type of procedure		
Roux-en-Y gastric bypass	319	80.2
Vertical/sleeve gastrectomy or, Sleeve	74	18.6
Don’t know/Others	5	1.3
Preoperative nutritional status		
Obesity I	16	4
Obesity II	155	38.9
Obesity III	227	57
Current nutritional status		
Low weight	1	0.3
Eutrophy	92	23.1
Overweight	147	36.9
Obesity I	102	25.6
Obesity II	45	11.3
Obesity III	11	2.8
Lowest BMI achieved		
Low weight	4	1
Eutrophy	145	36.5
Overweight	143	36
Obesity I	67	16.9
Obesity II	32	8.1
Obesity III	6	1.5

**Table 3:** Satisfaction with and distortion of body image and domains of eating behavior.

Variables	n *	%
Satisfaction with body image		
Dissatisfied/bigger silhouette	20	5
Satisfied	51	12.8
Dissatisfied/smaller silhouette	327	82.2
Body image distortion		
Absence of distortion	245	61.6
Mild distortion	93	23.4
Moderate distortion	43	10.8
Severe distortion	17	4.3
Domains of Eating Behavior		
Emotional eating (EE)	61	15.3
Eating disorder (ED)	19	4.8
Cognitive restriction (CR)	302	75.9
EE + ED *	3	0.8
EE + CR *	13	3.3

* In these cases, there was a tie for the predominant dominance.

**Table 4:** Frequencies and gross prevalence ratio of eating behavior domains according to distortion of and satisfaction with body image.

Variables	Domains of Eating Behavior ***											
Body image distortion *	Emotional eating (*p* = 0.001)				Eating disorder (*p* = 0.27)				Cognitive restriction (*p* = 0.001)			
	n	%	RP ^1^	IC95%	n	%	RP ^1^	IC95%	n	%	RP ^1^	IC95%
Absence	21	34.4	1		9	47.4	1		210	69.8	1	
Light	19	31.1	2.74	(1.4–5.4)	8	42.1	2.46	(0.9–6.6)	59	19.6	0.29	(0.2–0.5)
Moderate	16	26.2	6.32	(2.9–13.6)	1	5.26	0.62	(0.1–5.1)	23	7.64	0.19	(0.1–0.4)
Severe	5	8.2	4.44	(1.4–13.8)	1	5.26	1.63	(0.2–13.8)	9	2.99	0.19	(0.1–0.5)
Body image satisfaction **	Emotional eating (*p* = 0.336)				Eating disorder (*p* = 0.23)				Cognitive restriction (*p* = 0.064)			
	n	%	RP ^1^	IC95%	n	%	RP ^1^	IC95%	n	%	RP ^1^	IC95%
Dissatisfied/larger silhouette	2	3.3	1		1	5.26	1		17	5.7	1	
Satisfied	5	8.2	1.02	(0.2–5.8)	0		1	(0.0–0.1)	44	14.6	0.9	(0.2–3.9)
Dissatisfied/smaller silhouette	54	88.5	1.82	(0.7–4.8)	18	94.7	2.91	(0.4–22.3)	240	79.7	0.43	(0.2–1.0)

* Body Shape Questionnaire (BSQ). ** Silhouette Scale from Stunkard et al. [18] (1983). *** Three Factor Eating Questionnaire (TFEQ-R21). ^1^ PR = crude prevalence ratio.

## Data Availability

The data presented in this study are available upon request from the corresponding author, R.C.B.R.
